# Changes in bone density and bone turnover in patients with rheumatoid arthritis treated with rituximab, results from an exploratory, prospective study

**DOI:** 10.1371/journal.pone.0201527

**Published:** 2018-08-06

**Authors:** Gillian Wheater, Mohsen Elshahaly, Kamran Naraghi, Stephen P. Tuck, Harish K. Datta, Jacob M. van Laar

**Affiliations:** 1 Department of Biochemistry, The James Cook University Hospital, Middlesbrough, United Kingdom; 2 Institute of Cellular Medicine, Newcastle University, Newcastle-upon-Tyne, United Kingdom; 3 Rheumatology and Physical Medicine Department, Suez Canal University, Ismailia, Egypt; 4 Leeds Institute of Rheumatic and Musculoskeletal Medicine, University of Leeds, Leeds, United Kingdom; 5 Department of Rheumatology, The James Cook University Hospital, Middlesbrough, United Kingdom; 6 Department of Rheumatology and Clinical Immunology, University Medical Center Utrecht, Utrecht, The Netherlands; VU University Medical Center, NETHERLANDS

## Abstract

Data describing the effect of *in vivo* B cell depletion on general bone loss in patients with rheumatoid arthritis (RA) are limited. Given the pathogenetic role of B cells in RA, it is tempting to speculate that B cell depletion might have a beneficial effect on bone loss. We prospectively investigated the changes in bone mineral density (BMD), bone turnover, inflammation and disease activity before and after rituximab in 45 RA patients over a 12 month period, 36 patients of whom completed the study and were included in the analysis. There was no significant change in our primary endpoint; lumbar spine BMD after 12 months. However, we found a significant decrease in neck of femur (mean -0.017 g/cm^2^, 95% CI -0.030, -0.004 p = 0.011) and total femur BMD (mean -0.016 g/cm^2^, 95% CI -0.025, -0.007 p = 0.001). Additionally, there was a significant increase in procollagen type 1 amino-terminal propeptide (P1NP) and bone specific alkaline phosphatase (BAP); biomarkers of bone formation (median change from baseline to 12 months; P1NP 11.3 μg/L, 95% CI -1.1, 24.8 p = 0.025; BAP 2.5 μg/L, 95% CI 1.2, 3.6 p = 0.002), but no significant change in bone resorption or osteocyte markers. The fall in BMD occurred despite improvement in disease control. Post-menopausal women had the lowest mean lumbar spine, femoral and forearm BMD at baseline and after 12 months, additionally they had a higher level of bone turnover throughout the study. In conclusion, BMD was maintained at the lumbar spine and forearm, but fell at the femur sites. A high prevalence of vitamin D deficiency was observed and these patients had lower BMD and evidence of higher bone turnover.

## Introduction

Rheumatoid arthritis (RA) is the most prevalent inflammatory joint disease, in which B cells play an important role. Depletion of B cells by the anti-CD20 antibody rituximab is a highly effective treatment of RA, which is now well established [1]. The skeletal complications of RA consist of focal erosions of marginal and subchondral bone, juxta-articular osteoporosis and generalised bone loss with reduced bone mass [2]. Patients with established RA have an annual decrease of -3.9% and -2.5% of bone mineral density (BMD) at the lumbar spine (LS) and femoral neck respectively [3]. Additionally some studies have reported that the greatest reduction in BMD occurs at the foreram sites and that forearm BMD correlates with clinical features of disease activity and markers of bone turnover [4]. The main cause of periarticular osteopenia and local bone erosions is chronic inflammation of the synovial membranes, whereas several factors such as disease activity, female gender, older age, glucocorticoid use and decreased mobility are known to promote generalised bone loss with reduced bone mass in RA patients. However disease activity is the major predictor and is independent of the other factors [5]. Furthermore, RA patients are reported to have lower levels of total 25-hydroxyvitamin D (25OHD) and this is associated with increased disease activity and musculoskeletal pain [6]. Serum 25OHD is also negatively associated with DAS28 score, ESR, platelets, IL-17 and IL-23 and RA patients with osteoporosis and osteopenia have significantly lower levels of 25OHD than those with normal BMD [7].

Recent investigations have provided abundant evidence for an intricate interaction between the immune and skeletal systems [8], but the role of B cells in osteoclastogenesis is controversial [9]. Data describing the effect of *in vivo* B cell depletion on general bone loss in patients with RA are still limited. We have previously presented work demonstrating a change in bone turnover markers (BTMs) that suggests a potential favourable effect of rituximab on bone density [10]. We have therefore undertaken a preliminary exploratory, prospective study over 12 months to measure changes in bone density and to look at factors that may influence the outcome such as change in disease activity and vitamin D status.

## Materials and methods

### Patients

This study (EudraCT no. 2010-020499-50) was approved by the NHS Health Research Authority National Research Ethics Service (UK) Committee North East-Newcastle and North Tyneside 1 (REC reference no. 10/H0906/57) and was sponsored by South Tees Hospitals NHS Foundation Trust, UK. Recruitment took place in ten UK centres and patients were followed up for 12 months. Written consent was obtained according to the Declaration of Helsinki. Patients fulfilled the American College of Rheumatology (ACR) classification criteria 2010 for the diagnosis of RA and the UK National Institute for Health and Care Excellence (NICE) eligibility criteria for treatment with rituximab. Patients were excluded if they were less than 18yrs old, had previously received any B cell depleting agent or had been treated for osteoporosis. Calcium, vitamin D, corticosteroids, non-biological DMARDs and treatment for concomitant medical conditions were all continued throughout the study at the discretion of the treating physician. Rituximab was administered following the recommended protocol as a 1g intravenous infusion on days 1 and 15 in conjunction with intravenous methylprednisolone 100 mg. Patients who responded to the first rituximab course received a second course at 6 months unless they attained a state of low disease activity, as measured by DAS28< 3.2, in accordance with clinical practice. The primary outcome measure was change in LS BMD. The secondary outcomes were: change in mean total femur, mean neck of femur and mean forearm BMD, change in BTMs, inflammatory markers and DAS28, all assessed between baseline and 12 months.

### Bone mineral density measurements

BMD was measured at baseline and 12 months after the first rituximab course by dual-energy X-ray absorptiometry (DXA). Measurements were taken at LS (mean L2-L4), total and neck of femur on both sides and ultra-distal radius (RUD) forearms. Femoral neck, total femur and forearm BMD measurements were reported as the mean of the right and left sides. Two different DXA machines were used across the 10 centres, GE Lunar Prodigy (Lunar, Madison, Wisconsin, USA) and Hologic Discovery (Hologic, Waltham, Massachusetts, USA). However, the same machine was used at baseline and follow-up measurement for each patient. The inter-assay coefficient of variation (CV), measured using a local spine phantom, for the different centres were all less than 1.8%.

### Laboratory assessments

Patients were assessed at baseline prior to rituximab treatment and then every 3 months over a 12 month follow up period. Routine laboratory investigations were performed locally and clinical assessment of disease activity was undertaken using the 28-joint disease activity score and wide range C-reactive protein. Fresh whole blood samples were analyzed using a flow cytometer (FACS Canto II) to determine the numbers of CD19^+^ B cells in a subset of patients as per the study protocol. Fasting morning blood samples were collected into EDTA and serum separator tubes, the plasma and serum were separated within 60 mins and immediately stored at -80°C until analysis. The inter-assay CV was <5% for automated and <15% for the manual assays. All measurements were performed as per manufacturer’s instructions and in a centralized laboratory to reduce analytical variation.

#### Bone resorption markers

βCTx was quantified in plasma by electrochemiluminescent immunoassay (ECLIA) on the E411 analyser (Roche Diagnostics, Lewes, UK). Tartrate resistant acid phosphatase isoform 5b (TRAP-5b) was measured in serum by a manual enzyme-linked immunosorbent assay (ELISA) (Immuno-Diagnostic Systems Ltd, Bolden, UK).

#### Bone formation and osteocyte markers

P1NP was quantified in plasma by ECLIA on the E411 analyser. Bone specific alkaline phosphatase (BAP) was quantified in serum by chemiluminescence on the iSYS analyser (Immuno-Diagnostic Systems, Bolden, UK). Sclerostin (SCL) and dickkopf-related protein 1 (DKK-1) levels were measured in serum using a manual ELISA (Biomedica Group, 1210 Wien, Austria).

#### Inflammatory marker

Wide range CRP (CRP) was quantified in serum by a latex-enhanced immunoturbidimetric assay on the Advia 2400 analyser (Siemens Healthcare Diagnostics, Camberley, UK).

#### Total 25-hydroxy vitamin D and parathyroid hormone

25OHD was quantified in serum by chemiluminescence on the iSYS analyser. Laboratory reference range (Deficient <25; Insufficient 25–50; Adequate 50–75; Optimum >75 nmol/L) [11,12]. Parathyroid hormone (PTH) was quantified in plasma by ECLIA on the E411 analyser.

### Statistical analysis

The determination of sample size was based on the primary endpoint, change in LS BMD 12 months after the first rituximab course. Assuming the true change in LS BMD is ≥0.01 g/cm^2^ and that the DXA scan is reproducible with a standard deviation (SD) of 0.02 g/cm^2^; then we calculated that the study would have 80% power to detect a statistically significant difference (at the 5% significance level) if 33 patients were included in the final analysis based on a one sample t-test. To allow for a 20% dropout we estimated that we needed to recruit 42 patients. Continuous data were presented as means and SD or medians and range depending on the distribution. Categorical variables were displayed as absolute frequencies and percentages. The primary and secondary endpoints were investigated using a one sample t-test or Wilcoxon signed rank test if not normally distributed. The median percentage change from baseline across 4 visits (3, 6, 9 and 12 months) was calculated for BTM’s, inflammatory markers and disease activity. Spearman’s rank correlation coefficient (Rs) was used to correlate change in BMD with change in BTMs and inflammatory markers. Missing BMD or biomarker measurements were not imputed at any time point as we used a completer analysis approach. P values ≤0.05 were considered statistically significant. Statistical analyses were performed using STATA v12 (STATA Corp, Texas, USA).

## Results

### Demographic and clinical characteristics

**Fig 1:**
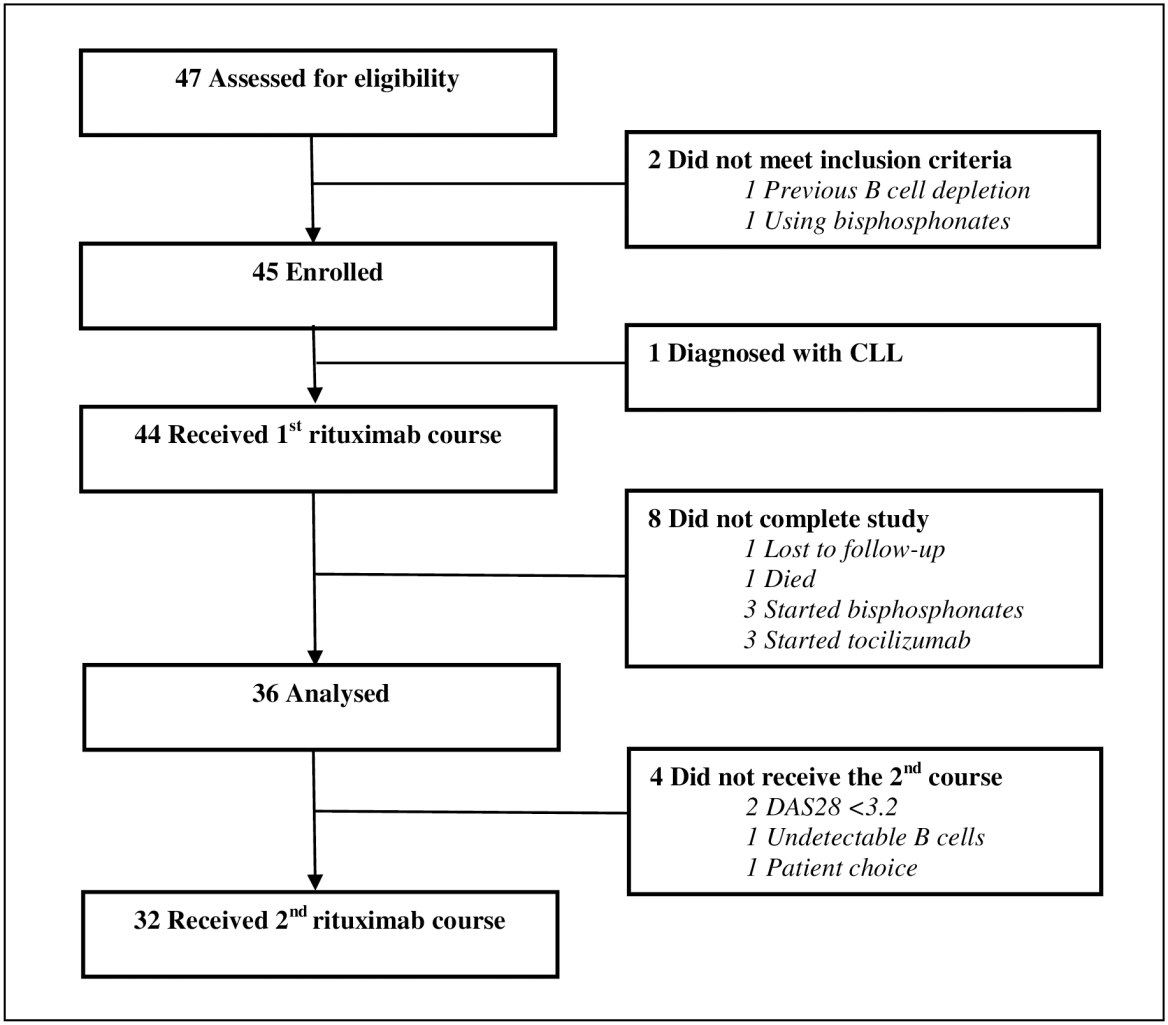
Consort flow diagram. Forty-seven patients were screened; 2 patients did not meet the eligibility criteria and so 45 were enrolled onto the study. One patient was subsequently diagnosed with chronic lymphocytic leukaemia therefore only 44 patients received the first course of rituximab. A further 8 patients did not complete the study and a total of 36 patients were included in the final analysis; of these only 32 received the second RTX course.

A total of 45 patients met the eligibility criteria and were enrolled into the study (Fig 1). One patient was diagnosed with chronic lymphocytic leukaemia (CLL) and excluded; therefore 44 patients received the first rituximab infusion. A total of 36 patients completed the 12 month follow up period and were included in the analysis; 32 of these patients received a second course of rituximab as per protocol and four patients did not (two patients had low disease activity (DAS28<3.2); one patient had undetectable B cells and one patient refused the second course). All patients were bisphosphonate naïve i.e. patients had not received bisphosphonates at any time prior to the start of this study. No fractures occurred during the study period. There was no significant difference in any baseline characteristic for those patients who completed the study (n = 36) compared to the 9 non-completers and the following analysis includes only those 36 patients who completed 12 month follow-up (Table 1). Briefly, 29 patients (81%) were female (23 of whom were post-menopausal) and seven (19%) patients were male. The mean age was 58.6 ±12.1yrs and the mean disease duration was 10.4 ±7.0yrs. Eighty-three percent (n = 30) and 76% (n = 25) were RF and ACPA positive respectively. Eleven patients were on steroids at baseline and a further 7 started steroids during the study. There was no significant difference between patients on steroids or not for change in BMD, inflammatory or bone biomarkers from baseline to 12 months. Thirty-nine percent (n = 14) of our patients had vitamin D deficiency defined as 25OHD levels below 25 nmol/L, only one of these patients was on a calcium and vitamin D supplement at baseline, a further four patients started on supplements during the course of the study. The numbers of CD19^+^ B cells were determined in a subset of 16 patients at each visit; all had values less than 0.01 x10^9^/L at 3 months and four patients had rapid reconstitution of their B cells at 6 months, while the others had long-term depletion.

**Table 1:** Baseline characteristics of the study patients.

Characteristics no. (%)	Completed study (n = 36)
**Age mean (sd), yrs**	58.6 (12.1)
**Male**	7 (19)
**Female**	29 (81)
**Pre-menopausal female**	6 (21)
**Post-menopausal female**	23 (79)
**Ethnicity White**	34 (94)
**Ethnicity Asian**	2 (6)
**Current smoker**	12 (33)
**Former smoker**	11 (31)
**Never smoked**	13 (36)
**Hydroxychloroquine**	1 (3)
**Leflunomide**	2 (6)
**Methotrexate**	22 (61)
**Sulfasalazine**	5 (14)
**Prednisolone**	11 (31)
**Calcium/ Vitamin D**	4 (11)
**BMI mean (sd), kg/m**^**2**^	29.4 (8.0)
**Disease duration mean (sd), yrs**	10.4 (7.0)
**RF positive**	30 (83)
**ACPA positive**	25 (76)
**DAS28-CRP median (range)**	5.74 (2.24, 8.01)
**HAQ mean (sd)**	1.92 (0.43)
**ESR median (range), mm/hr**	32 (2, 128)
**hs-CRP median (range), mg/L**	11.7 (0.4, 114.7)
**eGFR mean (sd), mls/min/1.73m**^**2**^	85 (25)
**PTH median (range), ng/L**	30.2 (14.1, 87.7)
**25OHD median (range), nmol/L**	36.1 (12.0, 141.0)
**LH median (range), U/L—females only**	30.1 (0.1, 70.9)
**FSH median (range), U/L—females only**	60.1 (0.6, 142.3)
**Testosterone median (range), nmol/L—males only**	12.4 (5.0, 26.0)
**SHBG median (range), nmol/L—males only**	49.7 (18.5, 80.0)
**βCTx median (range), ng/L**	423 (41, 1167)
**P1NP median (range), μg/L**	39.8 (10.9, 209.9)
**BAP median (range), μg/L**	17.2 (8.3, 37.3)
**TRAP5b median (range), U/L**	3.0 (1.1, 4.9)
**DKK-1 median (range), pmol/L**	47.9 (18.5, 119.4)
**SCL median (range), pmol/L**	53.4 (29.2, 88.2)
**Lumbar Spine L2-L4 BMD mean (sd), g/cm**^**3**^	1.171 (0.245)
**Lumbar Spine L2-L4 T score median (range)**	-0.4 (-2.6, 6.1)
**Lumbar Spine L2-L4 Z score median (range)**	0.4 (-2.7, 7.8)
**Mean neck of femur BMD mean (sd), g/cm**^**3**^	0.884 (0.140)
**Mean neck of femur T score median (range)**	-0.7 (-2.9, 1.0)
**Mean neck of femur Z score median (range)**	0.3 (-1.6, 1.9)
**Mean total femur BMD mean (sd), g/cm**^**3**^	0.944 (0.153)
**Mean total femur T score median (range)**	-0.5 (-2.7, 2.1)
**Mean total femur Z score median (range)**	0.3 (-1.8, 2.8)
**Mean radius UD BMD mean (sd), g/cm**^**3**^	0.381 (0.102)
**Mean radius UD T score median (range)**	-1.1 (-3.8, 8.6)
**Mean radius UD Z score median (range)**	-0.6 (-2.6, 10.4)

Continuous data are presented as means and standard deviation (SD) or medians and range, depending on the distribution of the data set. Categorical variables are displayed as absolute frequencies and percentages. Neck of femur, total femur and radius UD results are reported as the mean of both sides. 25OHD: Total 25-Hydroxy vitamin D; ACPA: Anti-cyclic Citrullinated Peptide Antibody; BAP: bone specific alkaline phosphatase; βCTx: β-isomerised carboxy terminal telopeptide of type I collagen; BMI: Body Mass Index; CRP: C-Reactive Protein; DAS28: Disease Activity Score using 28 tender and swollen joints; DKK-1: dickkopf-related protein 1; eGFR: estimated Glomerular Filtration Rate; ESR: Erythrocyte Sedimentation Rate; FSH: Follicle Stimulating Hormone; HAQ: Health Assessment Questionnaire; LH: Luteinising Hormone; P1NP: procollagen type 1 amino-terminal propeptide; PTH: Parathyroid Hormone; RA: Rheumatoid Arthritis; RF: Rheumatoid Factor; SCL: sclerostin; SHBG: Sex Hormone Binding Globulin; SJC: Swollen Joint Count; TJC: Tender Joint Count; TRAP5b: tartrate resistant acid phosphatase isoform 5b.

### Bone mineral density

**Fig 2:**
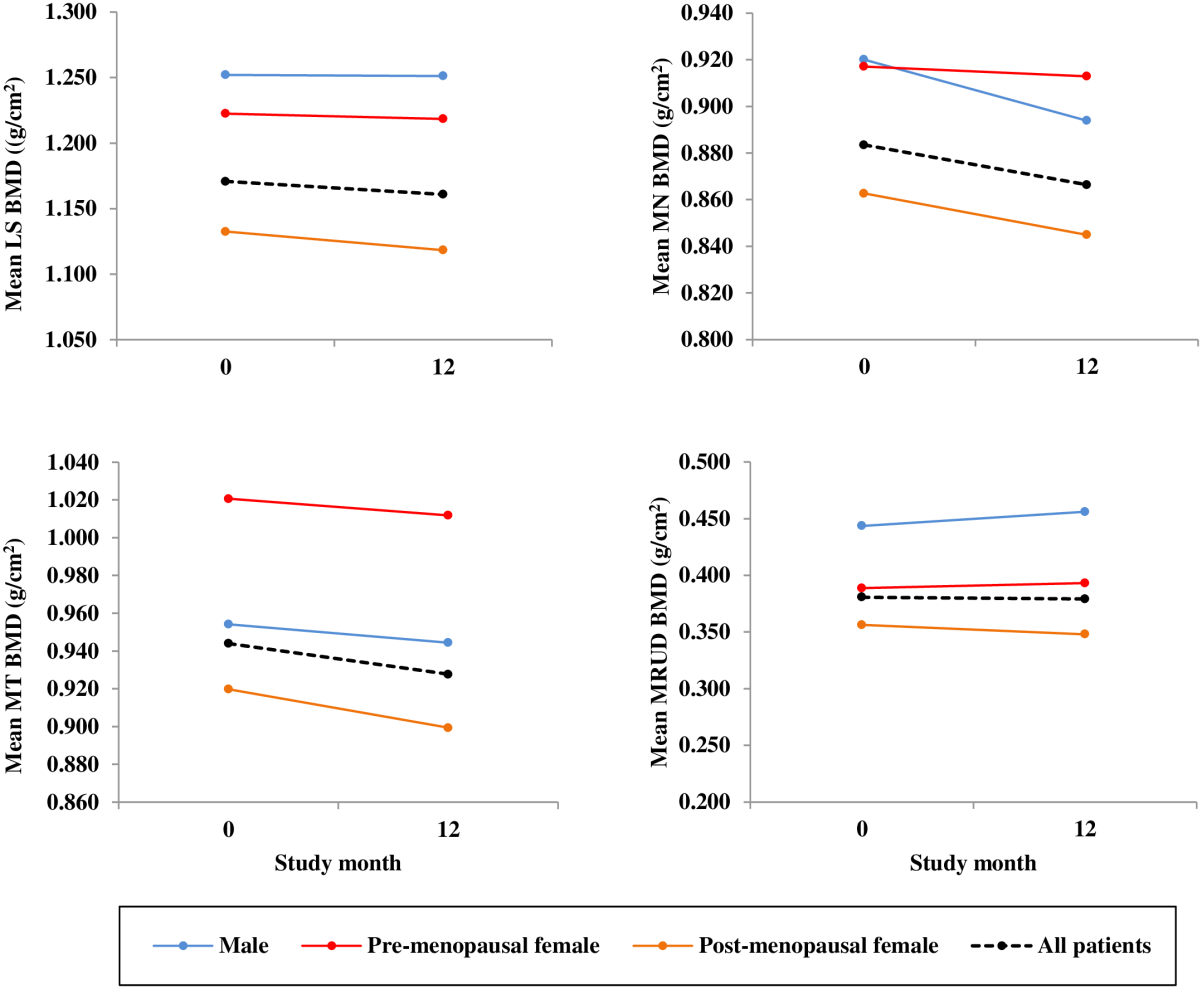
The effects of gender and menopausal status on mean bone mineral density. Bone mineral density (BMD) was plotted at lumbar spine; mean L2-L4 (LS BMD), also neck of femur (MN BMD), total femur (MT BMD) and ultra-distal radius (MRUD BMD) mean of both sides, at time 0 before the 1st rituximab infusion and after 12 months in patients who completed the study (n = 36). Patients were also stratified by gender and menopausal status (7 males, 6 pre-menopausal and 23 post-menopausal females).

There was no significant change in our primary endpoint; lumbar spine BMD after 12 months. However there was a significant decrease in mean neck of femur BMD (mean difference -0.017 g/cm^2^, 95% CI -0.030, -0.004 a decrease of -2.0%; p = 0.011) and mean total femur BMD (mean difference -0.016 g/cm^2^, 95% CI -0.025, -0.007 a decrease of -1.7%; p = 0.001) after 12 months (Table 2). Equally we found a significant decrease in both the left and right side femoral BMD. Six patients showed a decline in LS BMD greater than the least significant change (LSC) of 5%; 12 patients had a decrease in mean neck of femur BMD greater than 3%; 9 patients had a decline in mean total femur BMD greater than 3% and 12 patients had a mean ultra-distal forearm BMD decline of greater than 3%. Seven centres used the GE Lunar Prodigy Advance, and 3 centres used Hologic Discovery QDR. There was no significant difference between Lunar or Hologic machines for the change in lumbar spine, femoral or ultra-distal forearm BMD measurement from baseline to 12 months. As menopausal status is known to markedly affect bone turnover we also examined the data in gender- and menopausal status-specific manner. Mean BMD was plotted (Fig 2) at baseline i.e. before the 1st rituximab infusion and after 12 months in all patients who completed the study (n = 36) and by gender and menopausal status (7 males, 6 pre-menopausal and 23 post-menopausal females). We found that post-menopausal women had the lowest mean lumbar spine, femoral and forearm BMD at baseline and after 12 months.

**Table 2:** Change in mean bone mineral density from baseline to 12 months.

	Baseline mean (SD)	12 month mean (SD)	Mean change baseline to 12 months (95% CI)	p value change baseline to 12 months
**Lumbar Spine L2-L4 (n = 36)**	1.171 (0.245)	1.161 (0.250)	-0.010 (-0.029, 0.009)	0.302
**Mean Neck Femur (n = 35)**	0.884 (0.140)	0.867 (0.143)	-0.017 (-0.030, -0.004)	**0.011**
**Mean Total Femur (n = 35)**	0.944 (0.153)	0.928 (0.150)	-0.016 (-0.025, -0.007)	**0.001**
**Mean UD Radius (n = 32)**	0.381 (0.102)	0.380 (0.114)	-0.002 (-0.013, 0.010)	0.787

Bone mineral density (BMD) was measured in lumbar spine (n = 36) mean L2-L4. Also neck of femur (n = 35), total femur (n = 35) and ultra-distal radius (n = 31), the results were reported as mean of both sides. All measured at time 0 before the 1st rituximab infusion and after 12 months in patients who completed the study. Results were expressed as mean and standard deviation (SD) at baseline and 12 months, the mean change from baseline was assessed by paired t-test.

### Inflammation and disease activity

There was a significant reduction in inflammatory markers from baseline to 12 months (CRP median change -3.7 mg/L, 95% CI -10.3, 0.7 p = 0.014, a median percentage decrease over 4 visits of 21%; ESR median change -8 mm/hr, 95% CI -19, 1 p = 0.006, a median decrease of 20%) and disease activity (DAS28-CRP median change -0.84, 95% CI -1.64, -0.41 p<0.001, a median percentage decrease of 19%) following treatment with rituximab (Table 3). Eleven percent (n = 4) of patients achieved remission (DAS28 <2.6) and 25% (n = 9) at least low disease activity (DAS28 <3.2) after 12 months. Furthermore, based on EULAR DAS28 response criteria [13]; 39% (n = 14) of the study population experienced a good response (DAS28 improvement >1.2) and 58% (n = 21) at least a moderate response (DAS28 improvement between 0.6 and 1.2) over 12 months. We found no significant difference between patients achieving DAS28 <3.2 or those with DAS28 >3.2 for change in BMD or BTMs over 12months. However, there was a significant difference (p = 0.029) in DKK-1 levels between patients with good/moderate response (mean DKK-1 change -7.6 pmol/L, 95% CI -19.9, 5.3) and those with poor EULAR DAS28 response (mean DKK-1 change 8.7 pmol/L, 95% CI 0.01, 17.3) over 12 months.

**Table 3:** Change in median biomarkers from baseline to 12 months.

	Baseline median (range)	12 Month median (range)	Median change baseline to 12 months (95% CI)	p valuechange baseline to 12 months	Median % change baseline across all 4 visits (95% CI)
**βCTx (ng/L)** (n = 34)	423 (41, 1167)	384 (135, 1091)	-7 (-57, 59)	0.993	-8.0 (-23.0, 15.0)
**TRAP5b (U/L)** (n = 34)	3.0 (1.1, 4.9)	2.9 (1.5, 4.6)	0.1 (-0.3, 0.3)	0.638	0.0 (-7.0, 8.0)
**P1NP (μg/L)** (n = 34)	39.8 (10.9, 209.9)	48.8 (16.8, 125.0)	11.3 (-1.1, 24.8)	**0.025**	30.0 (3.0, 50)
**BAP (μg/L)** (n = 34)	17.2 (8.3, 37.3)	19.3 (10.9, 40.3)	2.5 (1.2, 3.6)	**0.002**	13.0 (4.0, 19.0)
**SCL (pmol/L)** (n = 34)	53.4 (29.2, 88.2)	55.7 (33.0, 89.0)	3.2 (-3.5, 8.6)	0.139	0.1 (-3.0, 8.0)
**DKK-1 (pmol/L)** (n = 34)	47.9 (18.5, 119.4)	51.5 (13.5, 150.8)	2.2 (-9.0, 9.7)	0.561	-2.0 (-10.0, 14.0)
**CRP (mg/L)** (n = 36)	11.7 (0.4, 114.7)	6.4 (0.2, 64.4)	-3.7 (-10.3, 0.7)	**0.014**	-21.0 (-49.0, 75.0)
**ESR (mm/hr)** (n = 32)	32 (2, 128)	17 (2, 43)	-8 (-19, 1)	**0.006**	-20.0 (-50.0, 25.0)
**DAS28 score** (n = 36)	5.74 (2.24, 8.01)	4.60 (1.38, 6.35)	-0.84 (-1.64, -0.41)	**<0.001**	19.0 (-27.0, -14.0)

Markers of bone resorption (βCTx: β-isomerised carboxy terminal telopeptide of type I collagen; TRAP5b: tartrate resistant acid phosphatase isoform 5b), bone formation (P1NP: procollagen type 1 amino-terminal propeptide; BAP: bone specific alkaline phosphatase), osteocyte markers (SCL: sclerostin; DKK-1: dickkopf-related protein 1), inflammatory markers (CRP: C reactive protein; ESR: erythrocyte sedimentation rate;) and disease activity (DAS28: disease activity score using 28 tender and swollen joints) were measured at baseline before the rituximab infusion and then at 3, 6, 9 and 12 months in all patients who completed the study. Results were not normally distributed and were expressed as median and range, median change from baseline to 12 months was assessed using Wilcoxon signed rank test and the median percentage change from baseline across all 4 visits was calculated.

### Bone turnover markers

**Fig 3:**
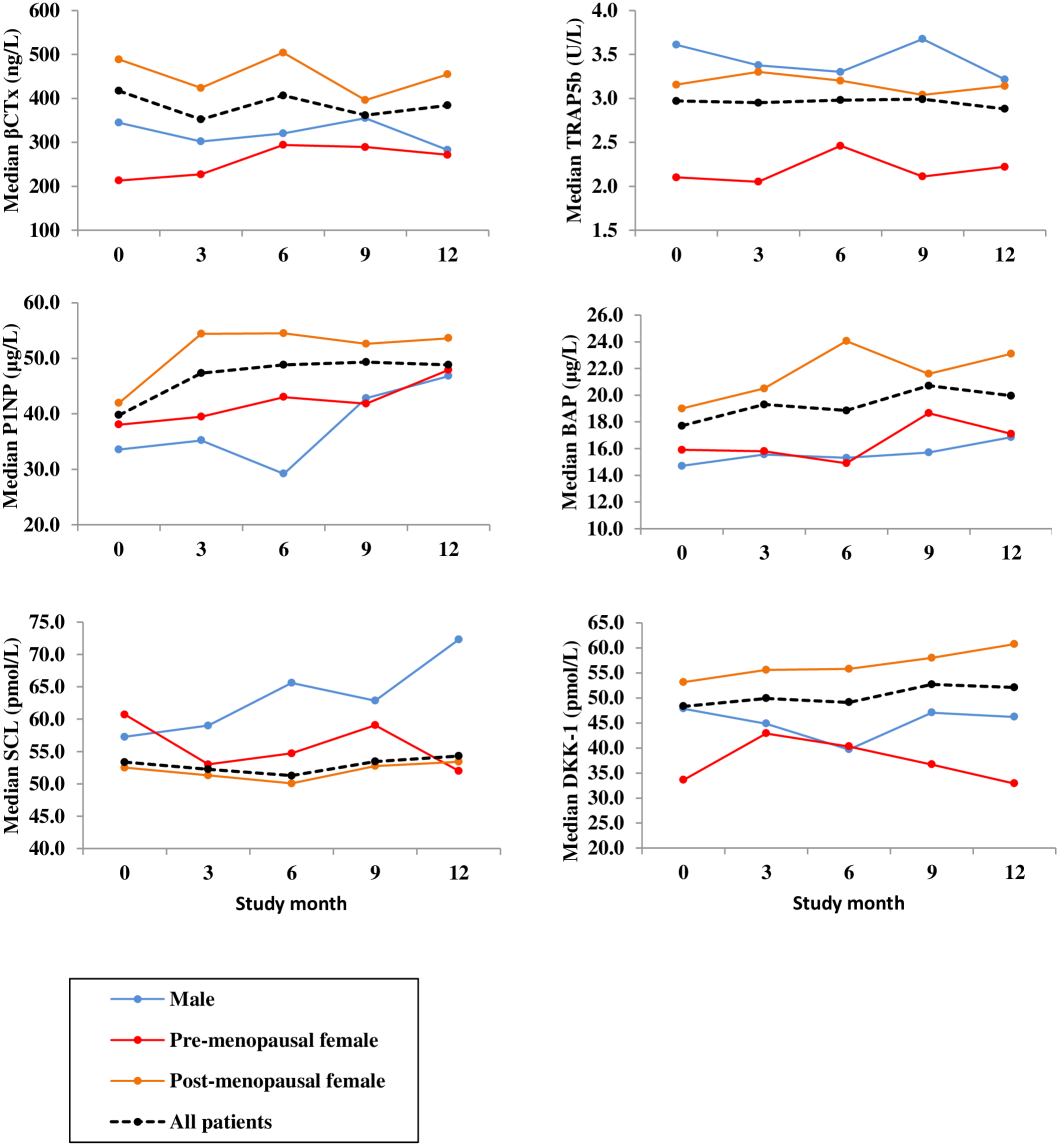
The effects of gender and menopausal status on median levels of biomarkers. Serial blood samples were taken at time 0 before the first rituximab infusion and then at 3 monthly intervals over one year, a second rituximab cycle was given if clinically indicated at 6 months. Median concentrations were plotted at each visit (all patients n = 34; post-menopausal females n = 22; pre-menopausal females n = 6; males n = 6) for the following biomarkers (y axis): β-isomerised carboxy terminal telopeptide of type I collagen (βCTx); tartrate resistant acid phosphatase isoform 5b (TRAP5b); procollagen type 1 amino-terminal propeptide (P1NP); bone specific alkaline phosphatase (BAP); sclerostin (SCL); dickkopf-related protein 1 (DKK-1).

We found a significant increase in bone formation over 12 months; P1NP (median change 11.3 μg/L, 95% CI -1.1, 24.8 p = 0.025; a median percentage increase over 4 visits of 30%)) and BAP (median change 2.5 μg/L; 95% CI 1.2, 3.6; p = 0.002; a median percentage increase of 13%). There was no significant change in bone resorption or osteocyte markers (Table 3). Median concentrations of BTMs were also plotted (Fig 3) for all patients (n = 34) at each study visit and we reviewed the data by gender- and menopausal status-specific manner (6 males, 6 pre-menopausal and 22 post-menopausal females). Post-menopausal women had the highest median concentrations of βCTx, P1NP, BAP and DKK-1 at baseline and throughout the 12 months, indicating a higher level of bone turnover.

### Effect of vitamin D concentration

**Fig 4:**
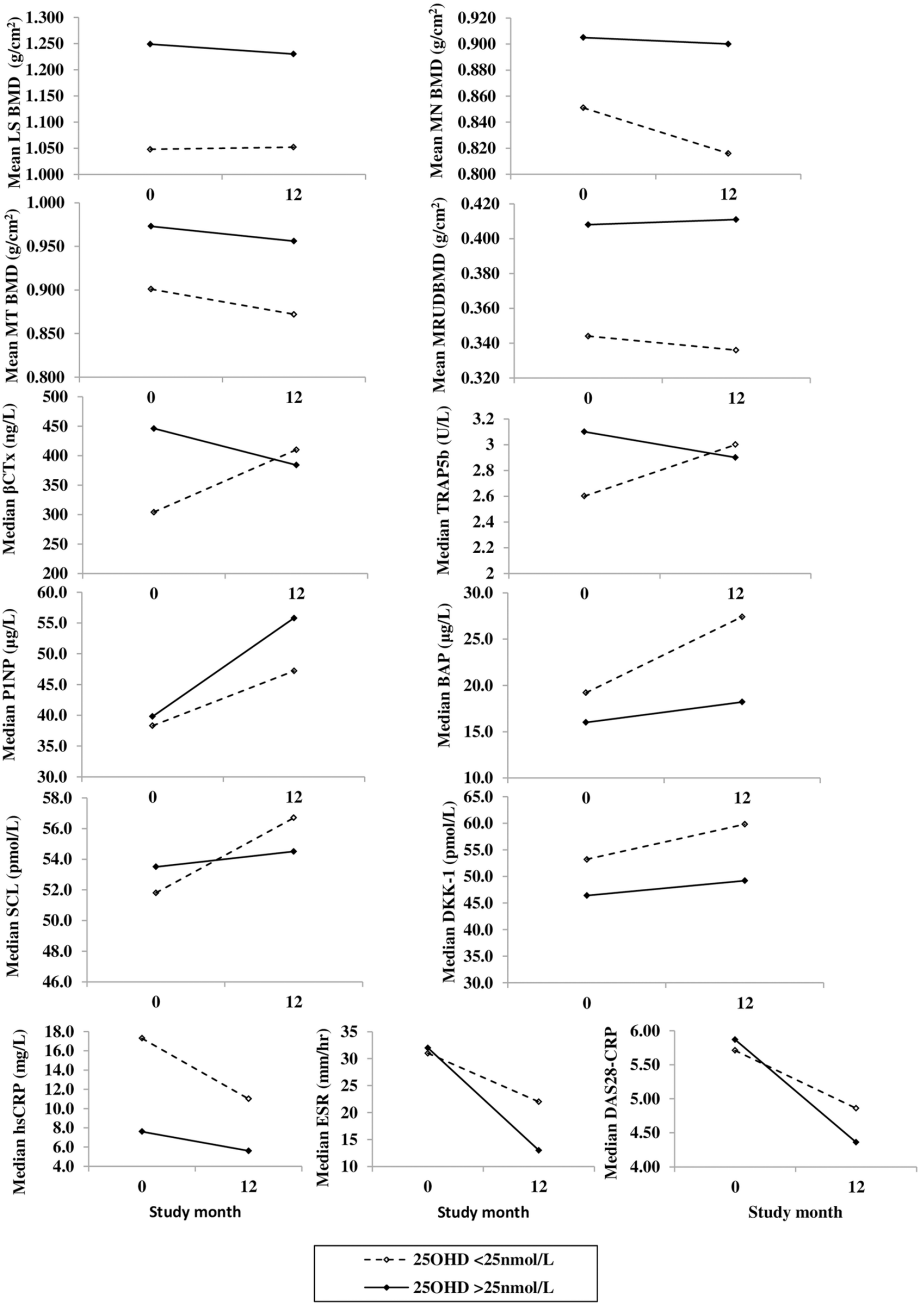
The effects of vitamin D on mean bone mineral density and median levels of biomarkers.

Thirty-nine percent (n = 14) of patients in our cohort were found to be vitamin D deficient, this included 29% (n = 2) of males, 33% (n = 2) of pre- and 43% (n = 10) of post-menopausal females. There was no significant difference (p = 0.274) in median 25OHD between males (69 nmol/L), pre- (36 nmol/L) or post-menopausal females (30 nmol/L). However, there was a significant difference in median PTH levels (p = 0.001) between males (19.8 ng/L), pre- (39.6 ng/L) and post-menopausal (33.0 ng/L) females. Total 25OHD was measured due to its effects on disease activity and its critical role in the maintenance of bone health, 25OHD levels could affect the primary and secondary endpoints and so results were stratified by 25OHD concentration; category 1 (n = 14) included levels below 25nmol/L; category 2 (n = 22) equal to or greater than 25nmol/L. BMD, BMT and inflammatory biomarkers were plotted at baseline and after 12 months by 25OHD category (Fig 4) We found that vitamin D deficient patients had the lowest mean lumbar spine, femoral and forearm BMD at baseline and after 12 months and median concentrations of bone resorption markers (βCTx, TRAP5b) increased from baseline to 12 months, whereas in vitamin D replete patients bone resorption decreased.

Results were categorized by 25OHD status; category 1 levels up to 24.9 nmol/L; category 2 levels greater than or equal to 25 nmol/L. Bone mineral density (BMD) was plotted for each 25OHD category at lumbar spine; mean L2-L4 (LS BMD), also neck of femur (MN BMD), total femur (MT BMD) and ultra-distal radius (MRUD BMD) mean of both sides, at time 0 before the 1st rituximab infusion and after 12 months in patients who completed the study (n = 36). A second rituximab cycle was given at 6 months if clinically indicated (n = 32). Additionally for each 25OHD category, median concentrations of the following biomarkers at time 0 and 12 months; β-isomerised carboxy terminal telopeptide of type I collagen (βCTx); tartrate resistant acid phosphatase isoform 5b (TRAP5b); procollagen type 1 amino-terminal propeptide (P1NP); bone specific alkaline phosphatase (BAP); sclerostin (SCL); dickkopf-related protein 1 (DKK-1); C-reactive protein (CRP); erythrocyte sedimentation rate (ESR); disease activity score using 28 tender and swollen joints (DAS28).

## Discussion

We prospectively studied the effects of B cell depletion using rituximab on bone density and bone turnover in patients with refractory RA over 12 months. We found no significant change in bone density at the lumbar spine; our primary endpoint, or ultra-distal forearm, however we did find a significant decrease in BMD at the femoral neck (-2%) and total femur (-1.7%). We also found a significant increase in bone formation in both P1NP and BAP, but no significant change in bone resorption or osteocyte markers after 12 months. Nevertheless, there was a significant reduction in inflammatory markers and disease activity, indicating that rituximab was effective in reducing the inflammation of RA in this patient cohort.

To our knowledge this is the first longitudinal study investigating the effects of rituximab on BMD and bone turnover markers. The fall in BMD at the hip sites was surprising given our previously published paper demonstrating a statistically significant 37% decrease in βCTx mirrored by a reduction in disease activity and an increase of 13% in P1NP, in sera of 46 RA patients 6 months after a single treatment course of rituximab, however no BMD data were collected [10]. There were differences in the study population between the two studies. Compared to our current study the patients in our earlier paper had lower disease activity and bone resorption at baseline and a greater improvement in bone turnover at 6 months. The present study included more postmenopausal females (79% compared to 58%) and notably a higher percentage of these women were current smokers (39% compared to 18%), above the national average quoted as 19% in 2013 [14]. Smoking may adversely influence the severity of RA [15] and the clinical effectiveness of rituximab [16], additionally tobacco reportedly increases bone resorption and affects bone mass [17].

The effects of tumor necrosis factor (TNF) blockers on bone have shown contrasting results; a study of 30 RA patients treated with TNF blockers in addition to methotrexate and prednisolone, reported an increase in LS BMD of 0.2% and 0.1% at the hip compared to a decrease in LS BMD of -0.8% and -0.6% at the hip in a control group of 10 patients who received only methotrexate and prednisolone [18]. Conversely; another study found a significant decrease in femoral neck BMD alongside an increase in BTMs in 54 RA patients treated with various TNF blockers over 6 months [19]. A recent review of the use of TNF inhibitors highlights this discordance [20]. However, results vary by study with regard to the magnitude of the observed change, the time points of the DXA scanning, the number and gender of patients and the concomitant use of prednisolone and/or anti-osteoporotic drugs [3,18–22].

Post-menopausal females in our study had the lowest mean lumbar spine, femoral and forearm BMD at baseline and after 12 months, reflecting the fact that bone loss is more rapid in post-menopausal women, partially due to lower circulating oestradiol levels [23]. None of our patients were treated for osteoporosis with bisphosphonates, calcitonin, strontium ranelate, denosumab or teriparatide prior to/ or during the study, yet BMD was maintained at lumbar spine, although there was a decrease in BMD at the femoral sites.

DKK1 and SCL are secreted osteocyte markers acting as inhibitors to the Wnt signalling pathway through binding to low density lipoprotein receptor-related protein 5 and 6 (LRP5/6) and hence blocking the Wnt effects on osteoblasts decreasing bone formation [24]. SCL inhibition in early human trials reportedly increases BMD and bone formation and decreases bone resorption [25,26]. However, inhibiting SCL in a human TNF-alpha transgenic mouse model of RA has been found to accelerate joint damage and the authors suggest that SCL therefore has a protective role in the presence of chronic TNFα–mediated inflammation [27]. It is interesting that treatment with rituximab had no significant effect on this particular BTM, additionally we found no significant variation in DKK-1 levels over 12 months.

Fourteen of our patients (39%) were classed as vitamin D deficient (25OHD <25 nmol/L), they had lower mean BMD at all sites at baseline and after 12 months compared to subjects with levels above 25 nmol/L. Additionally vitamin D deficient patients had an increase in bone resorption (median βCTx and TRAP5b levels) after 12 months, whereas patients with higher vitamin D levels had a reduction in bone resorption. These results are in keeping with a Chinese study which reported that serum 25OHD levels in 130 RA patients (95 women and 35 men), were lower in those with osteopenia and osteoporosis than in those with normal BMD [7]. A further study reported that higher 25OHD levels may prevent the occurrence of osteoporosis at the femoral neck but not lumbar spine [28] and one of the most recent factors found to be associated with autoimmunity is vitamin D deficiency [29]. However the methodology and definition of vitamin D deficiency varies widely between studies, many quoting a cut-off value as high as 50 nmol/L. In our cohort, although post-menopausal women had the lowest median 25OHD concentration we found no significant difference in median levels between males, pre- or post-menopausal women, so vitamin D deficiency may not explain why the post-menopausal women lost BMD more than pre-menopausal women or men. Vitamin D status is not normally investigated in patients presenting with RA, but our preliminary results suggest that vitamin insufficiency can influence outcome in terms of the response to treatment.

There are some limitations to our study; it was designed as a single treatment arm trial with no control group. The optimal design would be a double-blind randomized comparison with placebo. Patients with active RA would be expected to have continued bone loss and abnormal bone turnover until the disease activity had been adequately suppressed. It is therefore possible that the rituximab could have slowed the bone loss that was occurring, but this study would be unable to detect this without a control arm. But rituximab is an approved treatment for refractory RA and is already known to reduce disease activity [1], such a control arm would have had to be matched for disease activity and it would be unethical to have an untreated arm with that level of active disease. The duration of the study was also short at only 12 months; a longer follow-up of patients after subsequent treatment courses may have shown improvements in BMD, the relatively small numbers limits the power to detect smaller changes in variables and although all our patients had high disease activity and fulfilled the treatment criteria the group was heterogeneous, consisting of men, pre and postmenopausal women and different age groups.

In conclusion, the present study revealed that in a cohort of RA patients treated by rituximab BMD was maintained at the lumbar spine and UD radius forearm but fell at the hip sites. The effects of rituximab on BMD seems to be influenced by vitamin D status, gender, menopausal status, changes in disease activity and could also be confounded by the requirement for prednisolone. A larger study powered to take into account all these factors is required and this will necessitate that vitamin D insufficiency or deficiency be corrected from the start.

## Supporting information

S1 Table Study dataset. (XLSX)
